# Home-Based Transcranial Direct Current Stimulation (tDCS) to Prevent and Treat Symptoms Related to Stress: A Potential Tool to Remediate the Behavioral Consequences of the COVID-19 Isolation Measures?

**DOI:** 10.3389/fnint.2020.00046

**Published:** 2020-09-18

**Authors:** Luis Castelo-Branco, Felipe Fregni

**Affiliations:** Neuromodulation Center and Center for Clinical Research Learning, Spaulding Rehabilitation Hospital and Harvard Medical School, Boston, MA, United States

**Keywords:** transcranial direct current stimulation, tDCS, non-invasive brain stimulation, COVID-19, stress, stay-at-home orders, mental health, home therapies

## Introduction

The WHO has recommended self-isolation and social distancing measures for containing the coronavirus 19 (COVID-19) pandemic, and the Centers of Disease Control and Prevention (CDC) and the vast majority of countries have adopted it, with strategies varying in terms of degrees of containment (Wilder-Smith and Freedman, 2020). Despite the worldwide efforts in understanding and combating the disease, many uncertainties remain, including the precise rates of transmission and mortality, not to mention the proper treatment, leading to concerns and confusion regarding the necessary duration of the isolation measures. The possible need to adopt intermittent strategies of social distancing has also been raised, alternating periods of stricter and looser isolation (Kissler et al., 2020) until the discovery of a vaccine or efficient treatment is made.

Social isolation by itself leads to stress and negative mood (Brooks et al., 2020). The uncertainties mentioned above potentiate these effects, particularly in patients with a history of mental health disorders. Social isolation and loneliness are also associated with increased overall mortality (Steptoe et al., 2013), worse cardiovascular, and mental health outcomes (Leigh-Hunt et al., 2017) and cognitive decline (Evans et al., 2019). Furthermore, the recent Covid-19 outbreak has been associated with post-traumatic stress disorder (PTSD) symptoms, which were identified both in medical staff and the general population (Wang et al., 2020).

Numerous studies have demonstrated how non-invasive neuromodulatory techniques such as rTMS and tDCS can help prevent and ameliorate stress, with effects not only on emotion, cognition, and behavior but also on the cardiovascular (Makovac et al., 2017) and autonomic nervous systems (Schestatsky et al., 2013). Although non-invasive, repetitive TMS is expensive and not portable (the only portable option applies a very short train of pulses). Therefore, it is not appropriate during the context of social isolation since patients would have to leave their homes to receive the treatment. On the other hand, tDCS is potentially portable, and its feasibility in home-based settings has been previously studied with promising results regarding its safety and effectiveness (Martens et al., 2018; Brietzke et al., 2020).

Our objective in this short communication is to provide a comment on the potential tDCS application on preventing and treating stress-related symptoms during social isolation, while addressing the feasibility and efficacy of home-based tDCS.

## Tdcs and Stress: Treatment and Prevention of Related Symptoms

There is growing interest and studies investigating the effects of tDCS in animal models of stress and humans with chronic and acute stress. Animal models have shown reversal (Adachi et al., 2012) and prevention (Fregni et al., 2018) of chronic-stress induced pain after tDCS. In addition, tDCS was also able to decrease anxiety-like behaviors in rats submitted to neuropathic pain as a chronic stressor model (Marques Filho et al., 2016).

Interesting results in physiological surrogates of stress, namely heart rate variability (HRV) and salivary cortisol levels after tDCS treatment have been reported. Brunoni et al. (2013) showed that a single session of 1.5 mA anodal tDCS for 3 min targeting the left dorsolateral prefrontal cortex (DLPFC) led to an increase in high-frequency-HRV (HF-HRV) (Cohen's d = 0.77) and a decrease in salivary cortisol level (Cohen's d = 0.78) when compared to sham or cathodal stimulation in healthy individuals, reflecting effects in the autonomic nervous system and the hypothalamic pituitary adrenal (HPA) axis. An increase in parasympathetic and a decrease in sympathetic activity was also demonstrated in athletes by Montenegro et al. (2011) after 2.0 mA anodal stimulation for 20 min targeting the left temporal lobe, with an overall increase in HF-HRV compared to baseline measures (Cohen's d = 0.68).

Direct effects of tDCS on stress symptoms have also been recently published. Individuals such as healthcare workers, which are usually suffering from high levels of stress and even burnout, could benefit from preventive measures. When 1.075 mA tDCS stimulation of the right DLPFC was performed for 6–10 min in healthy individuals exposed to acute stress, there was less impairment of the working memory compared to sham or cathodal stimulation (Cohen's d = 0.62), showing the potential for the prevention of stress-induced mental disorders (Bogdanov and Schwabe, 2016).

On the other hand, patients who already developed psychiatric symptoms related to stress can also benefit from non-invasive brain stimulation. Anodal left DLPFC 1.0 mA tDCS has been shown as well to enhance the working memory when applied for 20 min once a week for 5 weeks in patients with the diagnosis of PTSD undergoing cognitive training programs compared to baseline, although the effects varied between subjects and were dependent on the performance test (Saunders et al., 2015). Another study demonstrated improved extinction-related processes in veterans with warzone-related PTSD when 2.0 mA anodal stimulation for 10 min was combined with fear-extinction therapy. tDCS targeted at the left ventromedial prefrontal cortex (vmPFC) during extinction-consolidation led to lower skin conductance reactivity compared to tDCS applied during extinction-learning (Cohen's d = 0.38) (Van't Wout et al., 2017).

**Figure 1:**
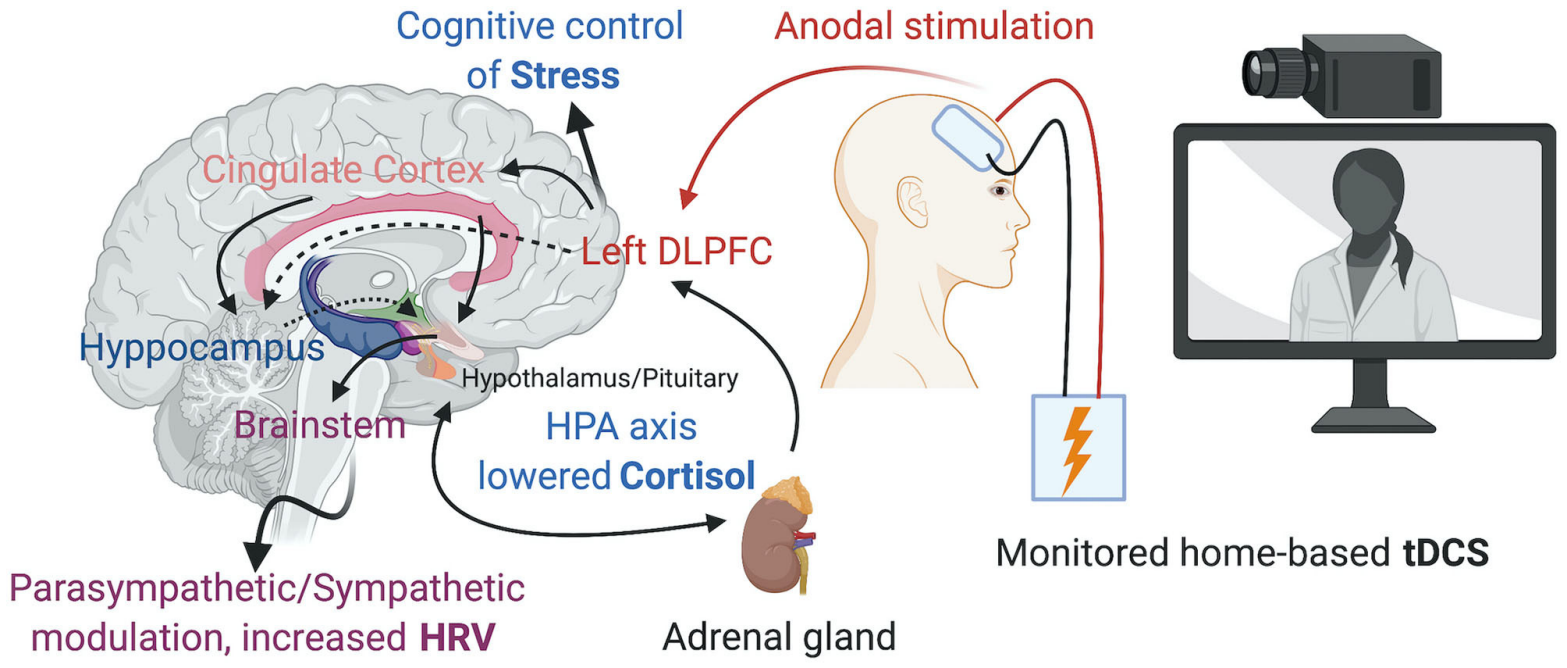
Home-based tDCS over the left DLPFC promoting the cognitive control of Stress, changes in the HPA axis and Parasympathetic/Sympathetic modulation. Created with BioRender.com.

The DLPFC has been the usual cortical target for tDCS to prevent and ameliorate the consequences of psychosocial stress (Figure 1). Carnevali et al. (2020) recently postulated that the effects of tDCS may involve both a cognitive control of stress and the autonomic system, involving predominantly parasympathetic (vagal) responses (Carnevali et al., 2020). Further research is needed, however, to determine whether tDCS could prevent the consequences of repeated or persistent exposure to stressful situations such as in the context of social isolation during a pandemic. In addition, the response to anodal 1.0 mA left DLPFC stimulation for 30 min can differ depending on individual anxiety traits (Sarkar et al., 2014), with individuals with high anxiety profiles improving performance in cognitive tests (Cohen's d = 0.82), as well as decreased cortisol levels (Cohen's d = 1.37) compared to sham stimulation, an effect that was not observed in subjects with low anxiety profiles. Therefore, assessing specific psychological traits at baseline could help determine which individuals would benefit more from the effects of tDCS.

Regarding emotional and affective states, tDCS has been well-studied for the treatment of patients with Major Depression, with improvement in depression scores and response rates supported by recent meta-analyses (Cohen's d = 0.74; Odds Ratio 2.44) (Brunoni et al., 2016) and (Hedge's g = 0.37; Odds Ratio 1.63) (Shiozawa et al., 2014). In heathy, non-depressed individuals, one study from Boggio et al. (2009) has shown that 3 min of anodal tDCS to the DLPFC significantly reduced ratings of unpleasantness in subjects exposed to distressing images compared to sham stimulation (Cohen's d = 0.88). Petrocchi et al. (2017) also demonstrated an increase in soothing positive affectivity (Cohen's d = 0.57) and an increase in HRV (Cohen's d = 0.26) after 15 min of 2 mA tDCS over the left temporal lobe compared to sham stimulation. However, other studies found no effect in improving the mood of healthy volunteers. It is worth mentioning that these negative studies explored the effect of a single (Morgan et al., 2014) or less than five sessions (Motohashi et al., 2013) of stimulation, and these very same studies raised the question of whether several consecutive sessions are needed to achieve mood-improving effects in non-depressed subjects. In addition, there have been no studies to our knowledge that applied tDCS as a preventive strategy to prevent future mood reductions.

## Feasibility of Home-based Tdcs

It is essential that the home-based tDCS device is specifically designed (Charvet et al., 2015) for this purpose, with safety measures that prevent the incorrect use of the equipment and guarantee the correct placement of the electrodes, since improper use of the device has been associated with skin burns. In order to mitigate such risk, most home-based tDCS devices have pre-programmed the intensity and duration of the stimulation and do not allow the patient to change them (Bikson et al., 2020), while others can be remotely controlled and adjusted by a technician.

Remote supervision using telehealth solutions (Riggs et al., 2018) are paramount to ensure safety and encourage adherence to the stimulation protocols, allowing the researcher to reassure the subject that mild and temporary sensations of tingling, itching, burning, or headache could occur during the stimulation, while also monitoring for unexpected or more severe adverse events, even if unrelated to the stimulation. With the current development of telehealth and ubiquitous use of remote visits, both patients and healthcare providers are becoming increasingly comfortable with this technology. The same data safety and cybersecurity measures used in regular remote clinical work can be used to monitor the use of home-based tDCS, safeguard the patient's privacy, and correct the use of the equipment. In the research setting, the use of electronic informed consent (eIC) is also becoming widely accepted to ensure the proper legal and ethical requirements are met.

Although it is a fairly simple technique with minimal risks associated with the electrical current delivery when done within pre-defined safety parameters, we do not encourage the use of any do-it-yourself stimulation devices. We strongly recommend closely monitoring and supervision, mainly due to the dangers of misuse and overuse.

To date, protocols that have used home-based tDCS have started with an in-person training session, either a home visit from a technician or a visit to the research facility by the subject. This was done to ensure that the individuals understood and properly used it (Kasschau et al., 2016). In the context of stricter social isolation situations such as a quarantine or complete lockdown, future protocols could potentially use solutions with all the instruction and orientation sessions done remotely, but that would require testing for feasibility.

## Discussion

Many studies have shown that the effects of tDCS are optimal when combined with behavioral strategies, such as exercise, cognitive training, mindfulness techniques, and Virtual Reality training. In this sense, the remote visit concomitant with the tDCS use would allow both monitoring and the delivery of these other therapies (Wright and Caudill, 2020) through web-based management systems (Chiesa and Serretti, 2009).

Can et al. (2019b) developed an automatic stress detection system that employs machine learning tools and concluded that physiological modalities are more accurate than self-reported perceived stress when analyzing real-world data. With the development of wearable devices and sensors, the assessment of physiological responses to the treatments could become routine (Can et al., 2019a), providing real-world data from patients in their own environment instead of measurements made in the artificial clinical laboratory setting.

It is worth mentioning that other more recent transcranial electrical stimulation techniques, such as alternating current (tACS), pulsed (tPCS), and random-noise (tRNS) use similar devices and therefore could be used at home, although, to the best of our knowledge, they have not been tested in this setting so far. One particular technique with the potential for home-based use that acts on the autonomic nervous system is the vagal nerve stimulation. This neuromodulation modality can be applied non-invasively either by transcutaneous stimulation of the cervical or auricular branches and can be particularly interesting when used in closed-loop systems (Gurel et al., 2020).

Exploring the relationship between non-invasive neuromodulation and the immune system in the context of a viral pandemic, although highly speculative, is also promising. While viral infections often lead to an important dysregulation of immune responses (Qin et al., 2020), neuromodulatory strategies that can down-regulate the excessive inflammation and its detrimental effects (Silverman et al., 2005) could show beneficial effects in the adjuvant treatment and even prevention of infection.

Finally, we believe that, in the context of COVID-19 social isolation, remote supervision could lead to benefits that are not directly related to the tDCS stimulation, providing support and eventual guidance for the isolated subjects. We understand that, if properly trained to do so, the research personnel, through the screen, could reinforce the importance of maintaining social isolation according to the health authorities.

## Author Contributions

LC-B: conceptualization and writing—original draft preparation. FF: supervision and writing—reviewing and editing. All authors contributed to the article and approved the submitted version.

## Conflict of Interest

The authors declare that the research was conducted in the absence of any commercial or financial relationships that could be construed as a potential conflict of interest.
